# The abilities of nitrogen-fixing bacteria to produce siderophores affect their promoting effects on cucumber growth and antiphytopathogenic fungus activities

**DOI:** 10.3389/fmicb.2026.1756337

**Published:** 2026-04-29

**Authors:** Er-Xing Wang, Jia Li, Ya-Ting Zhang, Lu-Rong Xu, Yan-Wen Xue, Yun-Peng Chen

**Affiliations:** 1 Department of Resources and Environment, School of Agriculture and Biology, Shanghai Jiao Tong University, Shanghai, China; 2 Asset Management and Shared Equipment’s Office, School of Agriculture and Biology, Shanghai Jiao Tong University, Shanghai, China; 3 Shanghai Yangtze River Delta Eco-Environmental Change and Management Observation and Research Station (Shanghai Urban Ecosystem Research Station), Ministry of Science and Technology, National Forestry and Grassland Administration, Shanghai, China

**Keywords:** antifungal activity, *gua*A gene, *Kosakonia radicincitans* GXGL-4A, nitrogen-fixing bacteria, plant growth, siderophore synthesis

## Abstract

**Introduction:**

Many nitrogen-fixing bacteria (NFB) can synthesize siderophores. However, the contribution of NFB siderophore synthesis to plant growth has not been evaluated, and its impact on NFB’s antifungal activity remains unclear.

**Methods:**

The type of siderophore synthesized by the nitrogen-fixing bacterium (NFB) *Kosakonia radicincitans* GXGL-4A was determined. The effects of NFB siderophore production on plant growth were examined by inoculating cucumber with GXGL-4A and the mutant Δ*gua*A (which does not produce siderophores). The antifungal activities of NFB cell-free fermentation broth on the mycelial growth of *Fusarium oxysporum*, *Fusarium proliferatum*, *Rhizoctonia solani*, and *Botrytis cinerea* were evaluated. Recovery of the siderophore synthesis ability of Δ*gua*A upon the addition of exogenous guanylate was measured.

**Results:**

The strain GXGL-4A produces carboxylate-type siderophores. The antifungal ability of Δ*gua*A was significantly reduced compared to that of GXGL-4A (*P* < 0.05). Compared with the group without NFB inoculation, GXGL-4A treatment significantly increased cucumber seedling height and plant dry weight (*P* < 0.05), but had no significant effect on root length and root dry weight (*P* > 0.05). There was no significant difference in seedling height between the experimental groups (*P* > 0.05). The siderophore production capability of Δ*gua*A could be partially restored via the addition of 2% (w/v) guanosine monophosphate to LB medium. NFB inoculation maintained a relatively stable, weakly alkaline rhizosphere (7.35–7.71), whereas the pH of the control group fluctuated more significantly (7.49–6.45). On the 3rd day after inoculation, the rhizosphere electrical conductivity of the Δ*gua*A treatment group (1,461 μs/cm) was significantly lower than that of the GXGL-4A treatment group (1,707 μs/cm) and the control group (2,973 μs/cm, *P* < 0.001). Compared with the GXGL-4A treatment group, the leaf chlorophyll content of the Δ*gua*A treatment group was significantly decreased on the 9th day after inoculation (*P* < 0.05), whereas there were no significant differences in root activity, soil dehydrogenase activity, and alkaline protease activity (*P* > 0.05).

**Discussion:**

The loss of siderophore synthesis ability in NFB leads to a significant decrease in their ability to promote plant growth and their own antifungal capacity. By utilizing NFB with high siderophore production capability, it is expected to play a greater role in sustainable agricultural production.

## 1 Introduction

Guanosine 5’-monophosphate (GMP) is formed by the condensation of phosphate with the hydroxyl group on the fifth carbon atom of the ribose of a nucleotide. It is one of the five nucleotides that make up nucleic acids and serves as the precursor molecule for guanosine tetraphosphate (ppGPP), guanosine triphosphate (GTP), cyclic guanosine monophosphate (cGMP), and guanosine diphosphate (GDP). GMP plays multiple crucial roles in biology, including serving as a component of DNA and RNA, participating in cellular energy transfer and metabolic regulation, and exerting numerous positive effects on human health. For example, the cyclic dimeric GMP (c-di-GMP) has been reported to positively regulate bacterial exopolysaccharide (EPS) amylovoran biosynthesis and biofilm formation in *Erwinia amylovora* Ea1189. Moreover, cellulose production, activated by cyclic di-GMP through BcsA and BcsZ, has been demonstrated to be a virulence factor and an essential determinant of the three-dimensional architectures of biofilms formed by *E. amylovora* Ea1189 (Castiblanco and Sundin, 2018). *Pseudomonas aeruginosa* can regulate its virulence factors in response to external environment triggers, and the signaling mechanism involves two-component regulatory systems and small molecules such as bis-(3’, 5’)-cyclic dimeric guanosine monophosphate (Bhagirath et al., 2018). Elevated levels of the second messenger c-di-GMP in Comamonas testosteroni enhanced biofilm formation and biofilm-based biodegradation of 3-chloroaniline (Wu et al., 2015). *Rhodospirillum centenum* utilizes 3’,5’-cyclic guanosine monophosphate (cGMP) as a messenger to regulate the development of desiccation-resistant cysts (Dong et al., 2015). In the food industry, it is primarily used as a food additive in the form of its sodium salt (disodium guanylate) as a flavor enhancer, ingredient in soy sauce, and raw material for monosodium glutamate, often used together with glutamate salts to enhance the umami taste of food (Li et al., 2025; Gu et al., 2023). In the field of medicine, GMP can improve the body’s immune system, support anti-tumor effects, promote growth, and regulate nutritional metabolism; therefore, it is a promising pharmaceutical raw material for development (Tadao et al., 2025). GMP can be synthesized both via the salvage pathway and *de novo* synthesis. The nucleotide biosynthetic enzyme GMP synthase (GMPS) mediates the final step of the *de novo* synthesis of guanine nucleotides, converting xanthosine 5’-monophosphate (XMP) into GMP. In this reaction, glutamine acts as an amido-N donor and is converted to glutamate (Reddy et al., 2014). GMPS has been identified as an attractive target as it is essential for virulence in the clinically prominent fungal pathogens *Aspergillus fumigatus*, *Candida albicans*, and *Cryptococcus neoformans* (Nguyen et al., 2022). It has been confirmed that inactivation of the riboswitch-controlled GMP synthase GuaA in *Clostridioides difficile* is associated with severe growth defects and reduced infectivity in a mouse infection model. These results highlight the significance of *de novo* GMP biosynthesis in *C*. *difficile* during infection, indicating that targeting guanine riboswitches with analogs may be a viable therapeutic strategy (Smith et al., 2021). GMPS participates in chromatin and gene regulation across multiple types of organisms and is highly expressed in various human malignancies (Wang et al., 2021). Based on the available literature, most investigations on GMPS focus on human healthcare. There are no reports on GMPS in agricultural areas, such as nitrogen-fixing bacteria (NFB) and plant biocontrol and growth-promoting bacteria.

Iron is an essential trace element for plant growth and development. It has a significant impact on plant growth, metabolism, and photosynthesis. Iron is a crucial component of photosynthetic pigments, playing a vital role in photosynthesis (Hasan et al., 2025; Zhang et al., 2025a). An iron deficiency can hinder chlorophyll synthesis and energy supply processes. Iron is essential to human health and plays a critical role in crop growth and development. Iron deficiency can impede overall plant growth (Nabeel et al., 2025). A recent study demonstrated that enhanced Fe transport via the phloem improved Fe availability by sequestering Fe ions and activating vacuolar transport pathways in rice shoots. Fe transport influenced the gibberellin (GA) catabolism. It modulated the cytokinin (CTK), jasmonic acid (JA), and ethylene (ETH) signaling to induce Fe deficiency response and promote Fe uptake (Lin et al., 2025). The role of iron in microorganisms is multifaceted, affecting not only basic life activities but also the structure and function of microbial communities (Li et al., 2025). Iron is a necessary element for synthesizing cellular components and is indispensable for maintaining enzyme activity, as it participates in the synthesis and maintenance of various enzymes. In microbial energy metabolism, iron plays a crucial role in energy storage and transfer. Iron also plays a role in regulating cell osmotic pressure. The availability of iron directly affects microbial growth, reproduction, and pathogenicity (Parmanand et al., 2019). Adequate iron supply enhances microbial infection capability, while iron deficiency restricts microbial growth (Sujata et al., 2025). Iron deficiency can alter microbial community structure, affecting species diversity and interactions (Jiang et al., 2024).

Siderophores are a class of low-molecular-weight compounds synthesized by microorganisms under conditions of low iron availability. They specifically chelate ferric ions (Fe^3+^), helping microorganisms acquire this essential nutrient. The synthesis and transport of siderophores are highly expressed under iron-deficient conditions. Siderophores can be classified into hydroxamate-type, catecholate-type, α-hydroxycarboxylate-type, and mixed-type. Siderophores play significant roles in promoting plant growth and disease control (Mariangela et al., 2025). In highly alkaline soils with severe iron deficiency, plants cannot fully resolve the deficiency on their own. Many plant-growth-promoting rhizobacteria (PGPR) produce siderophores that form highly stable chelates with Fe^3+^. Root reductases of the host plant do not readily reduce the trivalent iron in these chelates. Still, they can be directly absorbed, converted, and utilized by the plant, thereby serving as a pathway for iron nutrition (Parul et al., 2025). Siderophores effectively control the occurrence and development of plant diseases through various mechanisms, including competing for iron, inducing plant resistance, and directly inhibiting pathogenic bacteria (Wang et al., 2025; Mythileeswari et al., 2025; Yang et al., 2024). Therefore, siderophores, as a new type of biopesticide, have broad application prospects in plant disease control.

The biosynthetic pathways of siderophores include the non-ribosomal peptide synthesis (NRPS) pathway and NRPS-independent pathways. The biosynthesis of siderophores is regulated not only by genes but also by multiple factors, including bacterial culture conditions, iron ion concentration, siderophore receptors, iron signaling, and transport (Feng et al., 2022). The impact of environmental factors on bacterial siderophore synthesis has been extensively reported, but there is limited information on the biosynthetic regulation of nitrogen-fixing bacteria (NFB). In our previous work, a Tn5 transposon mutant library of the nitrogen-fixing bacterium *Kosakonia radicincitans* GXGL-4A was constructed, and mutant M246-2, which does not produce siderophores, was identified, confirming that the *gua*A gene regulates siderophore synthesis in GXGL-4A (Zhang et al., 2025b). However, the effects of the *gua*A gene deletion on NFB’s biological functions and agricultural applications remain unclear. Therefore, in this study, a cucumber vermiculite cultivation system was established, and the cucumber seedlings were respectively inoculated with the wild-type strain GXGL-4A and its *gua*A gene deletion mutant (Δ*gua*A) to reveal the impact of the two strains on cucumber growth and analyze the differences in their siderophore synthesis capabilities and antibacterial activities against plant pathogens.

## 2 Materials and methods

### 2.1 Nitrogen-fixing bacterial strains and vermiculite

The NFB *K*. *radicincitans* GXGL-4A was isolated from corn root (Sun et al., 2018). The deletion mutant of the *gua*A gene (Δ*gua*A) encoding glutamine-hydrolyzing guanosine monophosphate (GMP) synthase, which was found to have no siderophore production ability, was constructed by homologous recombination double exchange in our previous study. The strain GXGL-4A has been stored at the China General Microbiological Culture Collection Center (CGMCC) under the preservation number CGMCC 12588. Vermiculite (particle size 2–3 millimeters) was purchased from Pajiadi Home Decor Flagship Store on Alibaba Group’s Taobao e-commerce platform.

### 2.2 Evaluation of inhibitory activities of NFB strains on the growth of phytopathogenic fungi

The NFB strains GXGL-4A and Δ*gua*A were cultured overnight in 100 mL of LB medium at 37°C. The bacterial cells were harvested by centrifugation at 8,000 rpm for 5 min. The supernatant was filtered through a syringe filter (Aquo-system, 0.22 μm), and the sterile filtrate was collected in 50 mL plastic centrifuge tubes for determining the inhibitory activities of NFB strains on the growth of phytopathogenic fungi.

Four phytopathogenic fungi including *Fusarium oxysporum* Schl., a soil-borne pathogenic fungus distributed worldwide, which can cause wilt disease in various plants such as cucurbits, solanaceous crops, bananas, cotton and legumes, *Fusarium proliferatum* causing rice sheath rot syndrome, *Rhizoctonia solani* Kühn causing rice sheath blight, and *Botrytis cinerea* Pers. causing tomato gray mold disease were respectively cultured on a potato dextrose agar (PDA) medium for 4–5 d at 27°C, and then used to test of antimicrobial activity. In the preparation of the antifungal experiment culture plate, for the experimental groups (T groups), the melted PDA medium (about 50°C) was thoroughly mixed with the sterile filtrate solution of an NFB fermentation broth in a volume ratio of 2:1. For the control group (CK group), the filtrate solution was replaced by sterile LB medium.

The cultured fungal plugs were taken from PDA medium and inoculated onto a newly prepared PDA plate containing sterile fermentation broth of an NFB strain (one plug per plate). The fungal proliferation within 6 d was visualized, and the diameters of fungal lawns were recorded. The fungal plugs were inoculated three times per group, and the resulting data were used for statistical analysis. The inhibition rate of an NFB fermentation broth on the growth of a phytopathogenic fungus is calculated using the following formula: inhibition rate = (the fungal lawn diameter in the CK group- the fungal lawn diameter in the T group)/(the fungal lawn diameter in the CK group- the original fungal plug diameter) × 100%.

### 2.3 Determination of the types of siderophores produced by the NFB strain GXGL-4A

To determine the type of siderophores synthesized by the NFB strain GXGL-4A, corresponding experimental measurements were conducted. The bacterial cells of GXGL-4A were cultured in 15 mL of LB medium at 37°C for 12 h. Then, 1.5 mL of the bacterial culture was inoculated into 50 mL of SA medium (L-asparagine, 2 g/L; sucrose, 20 g/L; potassium dihydrogen phosphate, 1 g/L; magnesium sulfate, 1 g/L; pH 7) and grown at 30°C for 48 h. Finally, the bacterial cells were removed by centrifugation at 12,000 rpm for 10 min to obtain the SA fermentation supernatant. This supernatant was then filtered through a 0.22 μm filter membrane to get a sterile fermentation supernatant for subsequent experiments.

To determine whether the GXGL-4A strain synthesizes catechol siderophores, an Arnow’s test (Arnow, 1937) was performed. 1 mL of the supernatant from the bacterium GXGL-4A fermentation was mixed with 100 μL of 5 mol/L HCl and 500 μL of the reaction solution (10 g each of Na_2_MoO_4_ ⋅ H_2_O and NaNO_2_ dissolved in 50 mL of double-distilled water). After reaction, 100 μL of 10 mol/L NaOH was added, and then the solution was diluted to 5 mL with double-distilled water. Finally, the reaction solution was scanned over the full wavelength range using a HACH DR 5000 ultraviolet-visible spectrophotometer (Loveland, CO, United States) to confirm the presence of a distinct absorption peak at 510 nm.

A Shenker experiment was conducted to determine whether the strain GXGL-4A can produce carboxyl-type siderophore (Shenker et al., 1992). The copper sulfate solution (final concentration of 250 μmol/L) and 2 mL of acetate buffer (pH 4.0) were added to 1 mL of bacterial fermentation supernatant. A full-wavelength scan of this solution was performed to determine whether there is a distinct absorption peak in the range of 190–280 nm.

To confirm whether the siderophores produced by *K*. *radicincitans* GXGL-4A were hydroxamate type, a ferric chloride test was performed (Schwyn and Neilands, 1987). 1 mL of 2% ferric chloride solution was added to 0.5 mL of SA fermentation supernatant. A UV-Vis spectrophotometer (UNICO 2802) was used to scan the wavelength range of 420–450 nm to detect any significant absorption peaks.

### 2.4 Evaluation of the relative contents of siderophores produced by different NFB strains

A liquid CAS assay was used to quantify the relative contents of siderophores. Specifically, cell-free supernatant was obtained from 1 mL of SA bacterial culture by centrifugation at 5,000 rpm for 5 min (4°C) and filtration (0.22 μm pore size membrane). Then, 500 μL of cell-free supernatant was mixed with CAS assay solution at a 1:1 ratio and incubated at 25°C in the dark for 0.5 h. The SA culture medium serves as a blank control. Absorbance at 630 nm (OD_630_) was measured using a microplate reader SpectraMax^®^ i3x Platform (Molecular Devices, CA, United States) in a 96-well plate for both the cell-free supernatant (As) and the SA culture medium control (Ar). The production of siderophores can be quantified using the following formula: Relative content of siderophores (SU) = (Ar—As)/Ar × 100%. Here, “Ar” represents the absorbance of the blank SA culture medium at 630 nm, and “As” means the absorbance of the sample (cell-free supernatant) at 630 nm (Wang et al., 2016).

### 2.5 The effect of adding exogenous guanosine monophosphate on the production of siderophores

The siderophore production abilities of *K*. *radicincitans* GXGL-4A and its *gua*A gene deletion strain Δ*gua*A were assessed using the blue agar chrome azurol sulfonate (CAS) assay method (Schwyn and Neilands, 1987). The strains GXGL-4A and Δ*gua*A were inoculated into 100 mL of LB medium each, serving as two controls. Additionally, the strain Δ*gua*A was inoculated into LB medium containing 2% guanosine monophosphate [recorded as Δ*gua*A(+)] and cultured at 37°C for 72 h. Subsequently, 10 μL of the bacterial suspension was dropped onto the CAS detection plate, and incubated in an incubator (37°C, dark culture) for 12, 24, 36, 48, and 60 h, respectively. The diameters of the yellow siderophore halos were measured, and statistical analysis was conducted to determine the effect of adding 2% guanosine monophosphate on the production of siderophores by the strain Δ*gua*A.

### 2.6 Germination of cucumber seeds and inoculation with NFB strains in seedlings

The cucumber (*Cucumis sativus* L.) seeds were surface-disinfected by immersion in a 75% ethanol solution for 30 s, followed by thorough washing with sterile water. They were then soaked in a 1% sodium hypochlorite solution for 10 min. After thoroughly washing with sterile water, the processed seeds were placed in a 37°C incubator for germination. A total of 80 g of sterile vermiculite was loaded in a 1-L beaker, and four germinated cucumber seeds were sown in a beaker containing the vermiculite. About 1 week later, the cucumber seedlings at the two-leaf and one-heart stage were respectively inoculated with different NFB strains.

The strains GXGL-4A and Δ*gua*A were inoculated at 1% (V/V) into 200 mL of LB medium for overnight cultivation. Then, the bacterial cells were collected by centrifugation at 8,000 rpm for 5 min at 4°C, and then resuspended in sterile water. Centrifugation and resuspension were performed twice, and the cells were finally suspended in an equal volume of sterile water. Each cucumber seedling was inoculated with 5 mL of the bacterial cell suspension (10^8^–10^9^ CFU/mL) once every 3 days. Five replicates of each treatment were prepared, yielding a total of 5 treatments by bacterial inoculation. The cucumber seedling leaves, roots, and rhizosphere vermiculites were carefully sampled on the 3rd (Day 3), 6th (Day 6), and 9th days (Day 9) after inoculation and stored at –20°C for subsequent experimental determinations.

### 2.7 Determination of cucumber seedling biomass

The growth of cucumber plants treated with NFB strains was analyzed by determining the biomass parameters, including root length, seedling height, and the fresh weights of roots and seedlings. The statistical analysis was performed using GraphPad Prism software (version 10).

### 2.8 Assessment of pH value and electrical conductivity in the cucumber rhizosphere

Approximately 5 g of vermiculite from the cucumber rhizosphere was collected on Days 3, 6, and 9 after NFB treatment, and thoroughly mixed with 25 mL of sterile water for pH and electrical conductivity (EC) determination. During the measurements, the mixture was centrifuged at 8,000 rpm for 5 min to obtain the supernatant. At each sampling time point, three rhizosphere vermiculite samples were taken from each experimental group to prepare the supernatant for measurement. The pH and EC values of the cucumber rhizosphere were assessed by using pH Meter PHS-3G and Electrical Conductivity Meter (DDS-307A) (Shanghai Leici Scientific Instrument Co., Ltd., Shanghai, China).

### 2.9 Evaluation of leaf chlorophyll content in cucumber leaves

In the sterile vermiculite cultivation system, cucumber leaves were collected on days 3, 6, and 9 after the inoculation with NFB strains. The leaf nitrogen contents were measured using a TYS-4N portable plant nutrient tester (Beijing Jinkelida Electronic Technology Co., Ltd., Beijing City, China). Automatic calibration of the meter was performed under natural indoor lighting. Each measurement lasted 5 s, and the values were recorded. Each leaf was measured at three locations: the leaf tip, mid-leaf, and leaf base. The average value was then calculated as the relative chlorophyll content of that leaf. Statistical analysis was performed using GraphPad Prism9 software (version 9.3.1.471).

### 2.10 Determination of soil enzymatic activity in the cucumber rhizosphere

The vermiculite samples taken from the cucumber rhizosphere on Days 3, 6, and 9 were prepared for air drying according to the instructions provided in the manual accompanying the reagent kit from Suzhou Mengxi Biomedical Technology Co., Ltd. (Suzhou, China). The corresponding weight of vermiculite samples collected from the cucumber rhizosphere was used for determining soil enzymatic activity, as recommended by the kit manufacturer’s experimental procedures. The light absorption value at a specific wavelength was measured using a microplate spectrophotometer, SpectraMax i3x Platform (Molecular Devices, CA, United States). The activities of two soil enzymes, dehydrogenase (S-DHA) and alkaline protease (S-ALPT), were measured using soil enzyme test kits (catalog numbers: M1412A, M1418B) from Suzhou Mengxi Biomedical Technology Corporation (Ltd.) (Suzhou City, China). The experiment was performed according to the product instructions. Three replicate samples were set up in each group, and the results were used for statistical analysis.

### 2.11 Evaluation of cucumber root activity

0.5 g of cucumber root tips from each experimental group was placed into 15 mL test tubes, respectively, and immersed in 10 mL of a mixture of 0.6% 2,3,5-triphenyltetrazolium chloride (TTC) and phosphate buffer solution (pH 7.0). The tubes were then incubated in the dark at 37°C for 1 h. Subsequently, 0.5 mL of 2 mol/L sulfuric acid was added to terminate the reaction. The root tip was ground, and ethyl acetate was added to extract the red reaction product named TTF. The extract was then diluted to 5 mL, and the absorbance was measured at 485 nm. The relative activity of the root was calculated using the formula, TTC reduction rate = TTC reduction amount (μg)/root weight (g) × time (h). The TTC standard curve was prepared as follows. The concentration gradients of 0.0035, 0.007, 0.014, 0.021, and 0.028 μg/mL were used with TTF from Shanghai Caiyou Industrial Co., Ltd. (Shanghai, China). Ethyl acetate was used as the blank, and absorbance was measured at 485 nm. A standard curve was then plotted [Y = 29.607X + 0.0071, R2 = 0.9891. Y: absorbance value at 485 nm wavelength; X: TTF concentration (μg/mL)].

## 3 Results

### 3.1 Antifungal activity of the NFB strains GXGL-4A and Δ*gua*A

**FIGURE 1:**
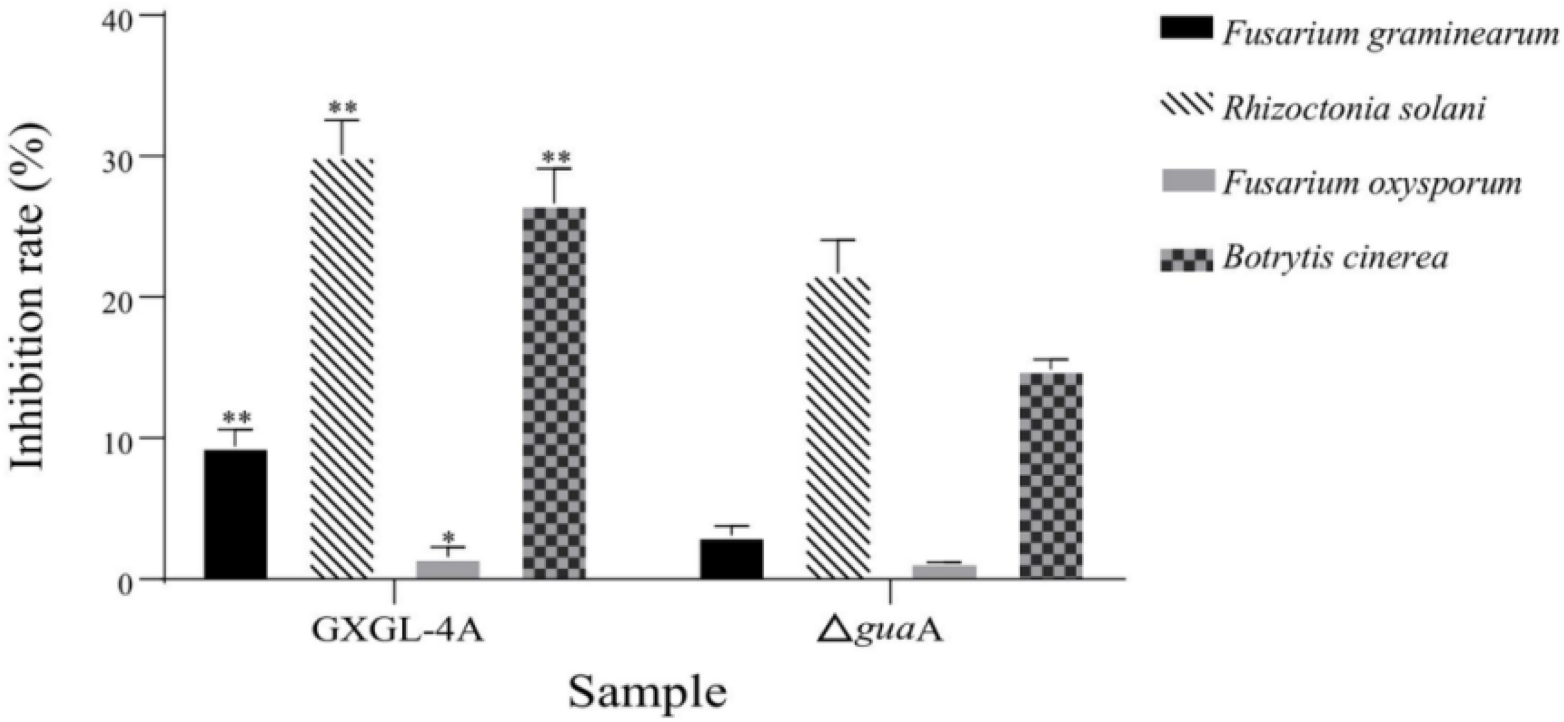
The inhibitory effect of the sterile culture supernatants from the wild-type strain GXGL-4A and its *gua*A gene deletion strain (Δ*gua*A) on the mycelium growth of four phytopathogenic fungi, including *Fusarium graminearum*, *Rhizoctonia solani*, *Fusarium oxysporum*, and *Botrytis cineerea*. The comparison of antifungal activity of two NFB strains against the same fungus is conducted by statistical analysis, and the significant differences are indicated by symbols “**” and “*” on the bar charts of the GXGL-4A treatment group, representing highly substanial (*P*< 0.01) and significant (*P*< 0.05) differences, respectively.

The filtered and sterilized fermentation broths of the NFB strains GXGL-4A and Δ*gua*A were used to test their abilities to combat phytopathogenic fungi. The results indicated that two NFB strains exhibited specific inhibitory effects on the growth of the tested fungi. The antifungal abilities of two NFB fermentation liquids showed an extremely significant difference against three phytopathogenic fungi (*P* < 0.01). Among them, the wild-type (WT) strain GXGL-4A fermentation broth had the best inhibitory effect on the *R*. *solani*, with an inhibition rate of 29.05%, and the inhibition rates for the tomato gray mold pathogen *B*. *cinerea* and the rice panicle rot pathogen *F*. *proliferatum* were about 25 and 9%, respectively. The sterile fermentation broth of two NFB strains has no significant inhibitory effect on the growth of *F*. *oxysporum*. In a word, deletion of the *gua*A gene in the genome of *K*. *radicincitans* GXGL-4A could significantly reduce its inhibitory activity against phytopathogenic fungi (Figure 1).

### 3.2 Determining the type of siderophores produced by *K*. *radicincitans* GXGL-4A

**FIGURE 2:**
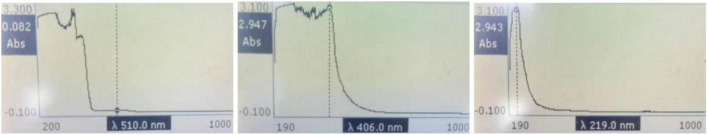
Detection of siderophore types. Catechol-type (absorbance peak at 510 nm) and hydroxamic acid-type (absorbance peak at 406 nm) siderophores were not detected. Carboxylate-type siderophore (absorbance peak at 219 nm) was identified.

The results of the Arnow’s test revealed that there was no absorption peak at the wavelength of 510 nm in the reaction solution, indicating that the NFB strain GXGL-4A does not produce catechol-type siderophores. Similarly, through the iron (III) chloride experiment, a distinct absorption peak in the range of 420 nm to 450 nm was not identified in the reaction solution, suggesting that the strain GXGL-4A does not produce hydroxamate-type siderophores. Eventually, the siderophore type was determined by Shenker test. A distinct absorption peak was detected at a wavelength of 219 nm, indicating that *K*. *radicincitans* GXGL-4A could produce an α-hydroxycarboxylate-type of siderophore (Figure 2).

### 3.3 Quantitative analyses of siderophore production in NFB strains

**FIGURE 3:**
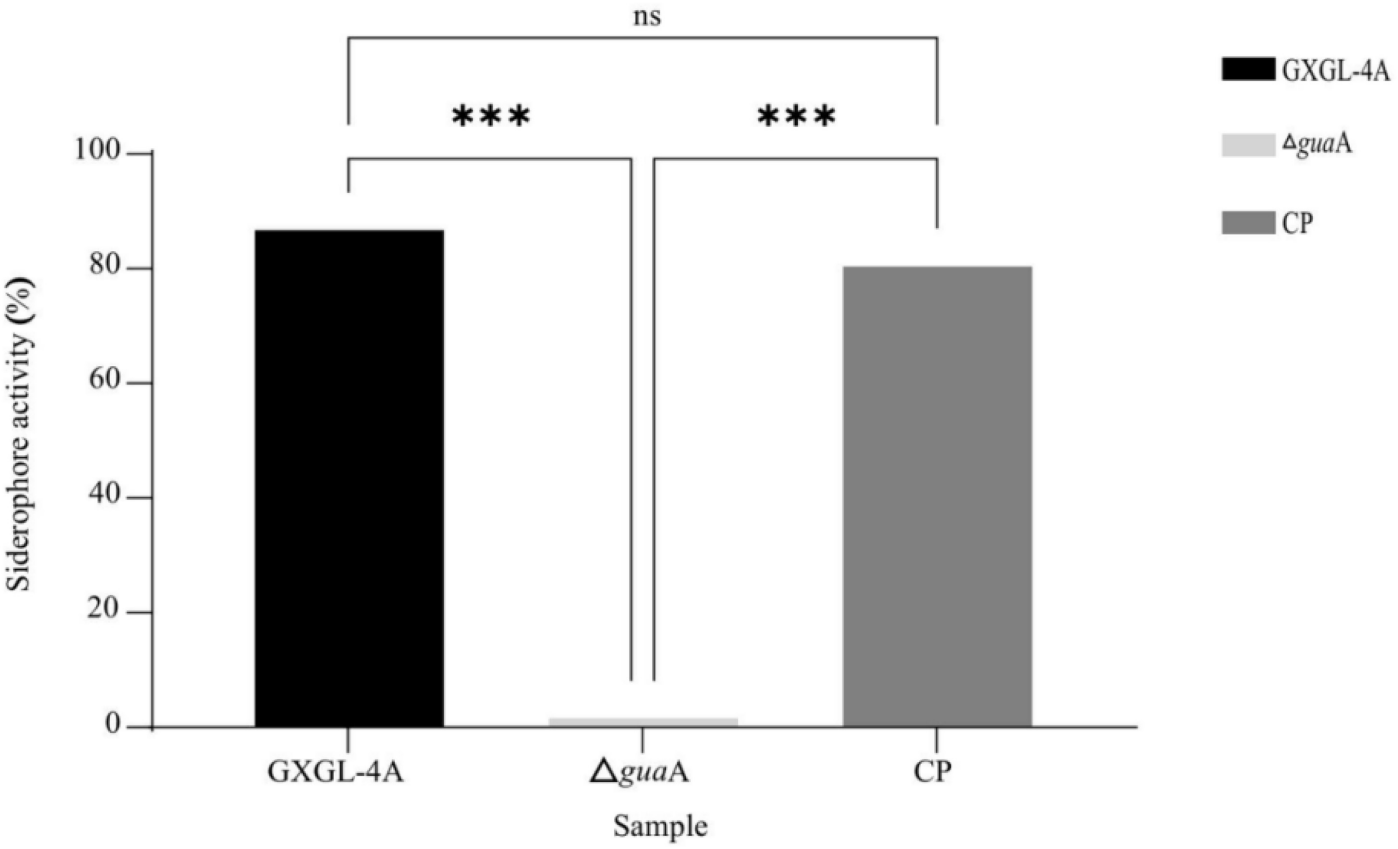
The content of Carboxylic acid-type siderophore produced by the wild-type strain GXGL-4A, Δ*gua*A, and the function-complementation strain of the guaA gene (CP). ns: no significant difference (*P* >0.05), ***: extremely significant differences (*P*< 0.001).

To reveal the effect of *gua*A gene deletion on siderophore production capability in *K*. *radicincitans* GXGL-4A, the relative siderophore contents in the mutant Δ*gua*A and the corresponding complementation strain (referred to as CP) were determined using liquid CAS assay. The experimental results showed that the *gua*A gene knockout strain Δ*gua*A failed to produce siderophore. The *gua*A gene functional complementation strain CP restored its normal ability to biosynthesize siderophore, indicating no significant difference in siderophore production compared to the wild-type strain GXGL-4A (Figure 3). Based on the above research results, it can be concluded that the *gua*A gene in *K*. *radicincitans* GXGL-4A is functionally related to siderophore production.

### 3.4 The effect of exogenous guanosine monophosphate on siderophore synthesis in the strain Δ*gua*A

**FIGURE 4:**
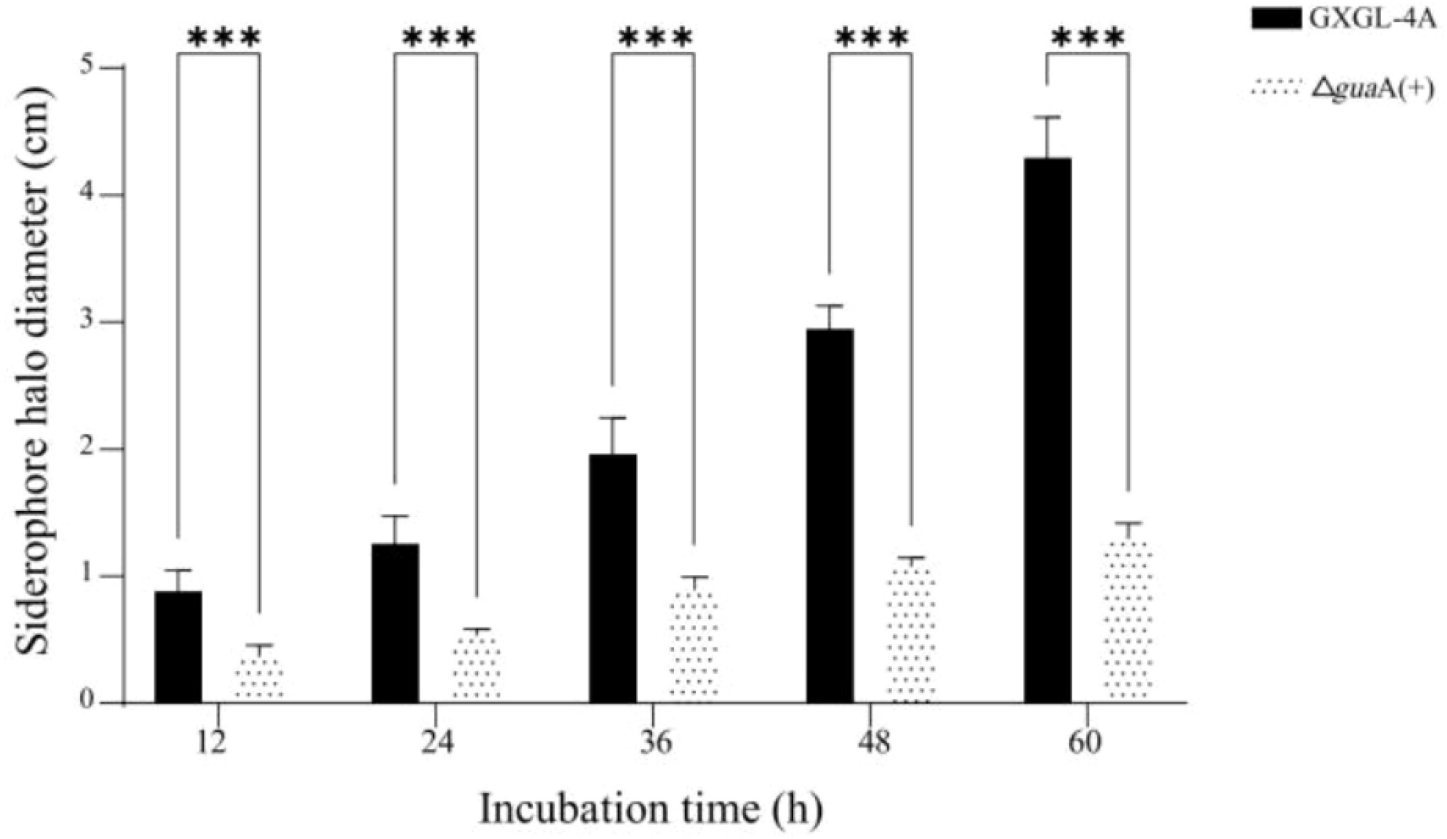
The diameters of the siderophore halo produced by the tested NFB strains on CAS-agar plates. Δ*gua*A(+): The *gua*A gene deletion strain Δ*gua*A was cultured in LB medium supplemented with 2% final concentration of guanylate. ***: extremely significant difference (*P*< 0.001).

The *gua*A gene deletion mutant Δ*gua*A was determined to produce siderophores—iron-binding compounds on CAS agar detection plates after a 72-h culture in LB medium containing 2% guanosine monophosphate by mass. Yellow halo zones around the Δ*gua*A colonies were visualized as early as 12 h after incubation in an incubator, and the halo zones continued to expand with the extension of incubation time. Although the siderophore production capability detected at each testing time point (12–60 h) during the experiment was still significantly lower than that of the wild-type strain GXGL-4A, the *gua*A gene deletion mutant Δ*gua*A undoubtedly restored the siderophore synthesis ability to some extent after the addition of exogenous guanosine (Figure 4).

### 3.5 EC and pH changes in the cucumber rhizosphere after inoculation with NFB strains

**FIGURE 5:**
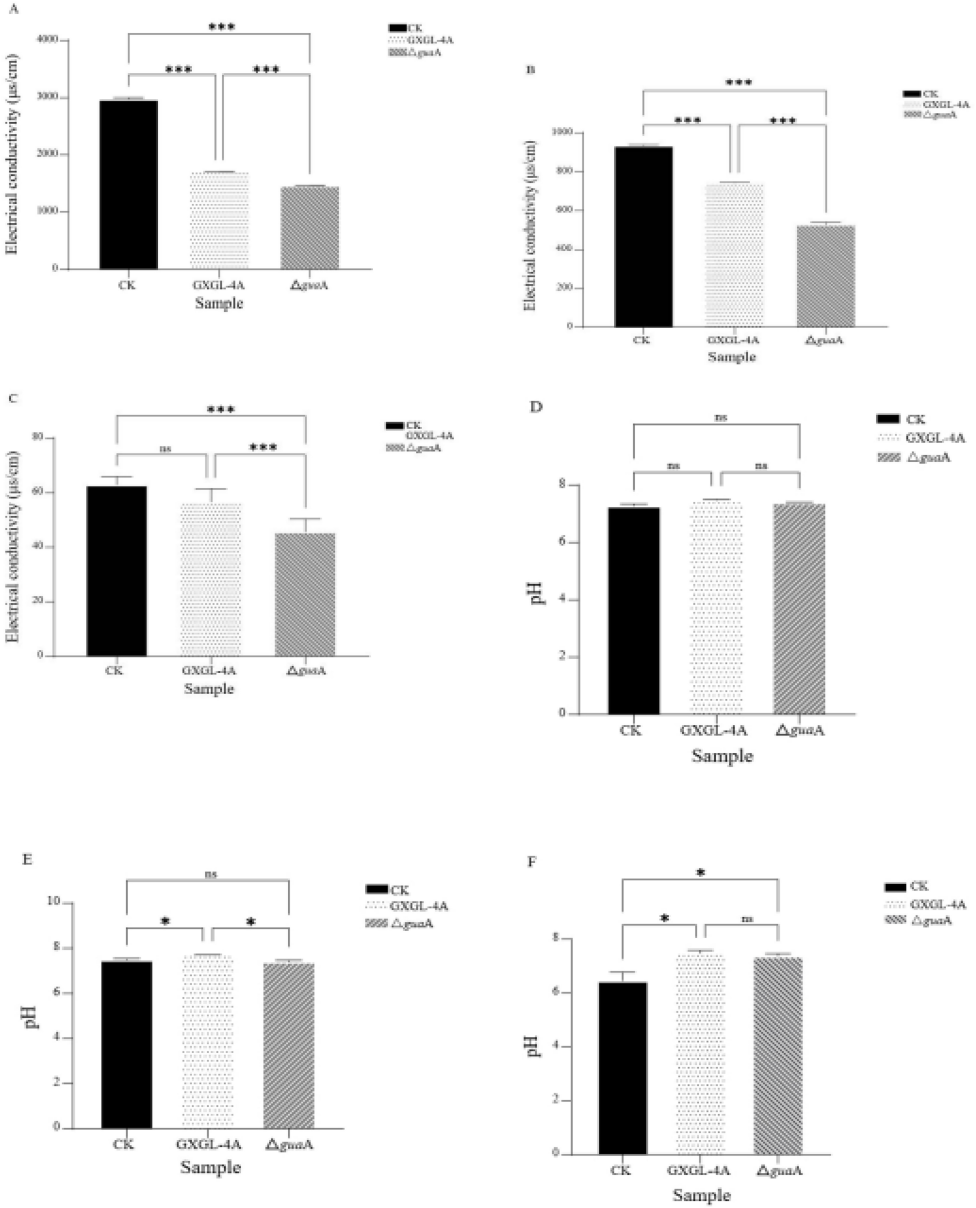
Evaluation of the electrical conductivity (EC, **A–C**) and pH **(D–F)** values in the cucumber rhizosphere environment. (A–C): The EC values of cucumber rhizosphere detected on Day 3 **(A)**, Day 6 **(B)**, and Day 9 **(C)** after inoculation with the NFB strains. **(D–F)**: The pH values of the cucumber rhizosphere detected on Day 3 (**D**), Day 9 (**F**) after inoculation with the NFB status. ***: extremely significant differences (*P*< 0.001). ns: no significant differences (*P* > 0.05). *: significant difference (*P <* 0.05).

On the 3rd and 6th days after inoculation (i.e., Days 3 and 6), the EC values of the NFB treatment groups were very significantly lower than those of the blank control group (CK group) (Figures 5A,B; *P* < 0.01). By the 9th day after inoculation, the EC value of the GXGL-4A-treated group was extremely significantly higher than that of the Δ*gua*A treatment group (*P* < 0.01). Still, there was no significant difference compared to the control group (Figure 5C; *P* > 0.05). Throughout the entire measurement period, the EC values of the three experimental groups showed a clear downward trend, with the CK group’s EC value decreasing from 2973 μs/cm on Day 3 to 62 μs/cm on Day 9. The two NFB treatment groups declined from 1,707 to 56 μs/cm and from 1,461 to 49 μs/cm, respectively. Furthermore, the detection results revealed that after inoculation with the NFB strains, the cucumber rhizosphere maintained a relatively stable, weakly alkaline pH, ranging from 7.35 to 7.71. In contrast, the control group (without bacterial inoculation) kept a low pH throughout the early (pH 7.27, Day 3) to mid-phase (pH 7.49, Day 6) of the experiment (Figures 5D,E). Still, it turned weakly acidic (pH 6.45) by the end of the experiment (Day 9, Figure 5F).

### 3.6 Relative chlorophyll contents in cucumber leaves after inoculation with NFB strains

**FIGURE 6:**
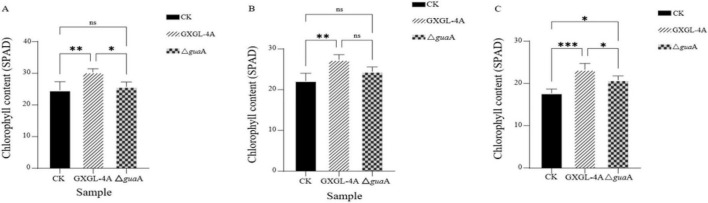
Relative chlorophyll contents in cucumber leaves. **(A)** The chlorophyll contents on Day 3 after inoculation with NFB strains. **(B)** The leaf chlorophyll contents on Day 6 after inoculation with NFB strains. **(C)** The leaf chlorophyll contents on Day 9 after inoculation with NFB strains. ns: There is no significant difference in cucumber leaf chlorophyll contents between two groups at the level of *P* = 0.05. *: There is a significant difference in leaf chlorophyll content between the two groups at *P* = 0.05. **: There is no significant difference in leaf chlorophyll contents between two groups at the level of *P* = 0.01. ***: There is a significant difference in leaf chlorophyll content between two groups at the level of *P* = 0.001. CK: The cucumber seedlings without NFB treatment. Δ*gua*A: The cucumber seedlings inoculated with the *gua*A gene deletion strain Δ*gua*A.

On the third day after inoculation with NFB strains (Day 3), the leaf chlorophyll contents of cucumber in the GXGL-4A treatment group were extremely significantly higher than those of the control group (without NFB inoculation) (*P* < 0.01). However, there was no significant difference between the group inoculated with the *gua*A gene deletion strain (Δ*gua*A) and the control group (Figure 6A). On Day 6, the leaf chlorophyll content in the GXGL-4A treatment group was still extremely significantly higher than that of the control group (*P* < 0.01). Still, at this time, there was no significant difference between this group and the Δ*gua*A treatment group (*P* > 0.05) (Figure 6B). On Day 9, the leaf chlorophyll contents in both NFB treatment groups were significantly higher than those of the control group (*P* < 0.05). Furthermore, it was noticed that chlorophyll content in the GXGL-4A treatment group was significantly higher than that of the Δ*gua*A treatment group (Figure 6C; *P* < 0.05).

### 3.7 The differences in cucumber biomass after inoculation with NFB strains

**FIGURE 7:**
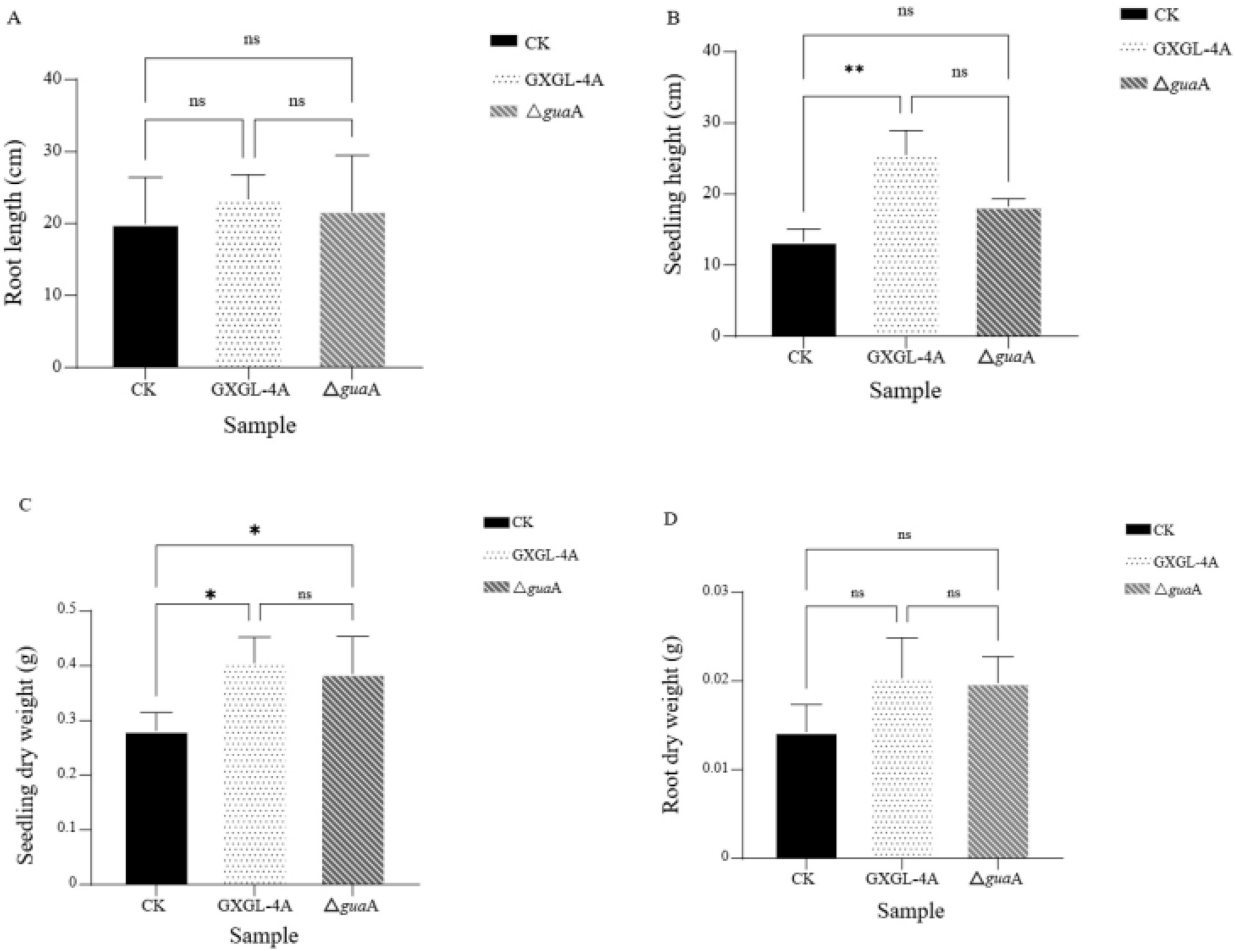
Biomass measurement on the 9th day (Day 9) after inoculation with the NFB strains. **(A)**: root length (cm). **(B)**: seedling height (cm). **(C)**: seedling dry weight (g). **(D)**: root dry weight (g).ns: There is no significant difference in this biomass parameter between two groups (*P* > 0.05). *: There is a significant difference in this biomass parameter between two groups at the level of *P* = 0.05. **: There is a significant difference in this biomass parameter between two groups at *P* = 0.01. CK: The cucumber seedlings inoculated with the *gua*A gene deletion strain Δ*gua*A.

On the 9th day after inoculation (Day 9), the seedling height and root length of each treatment group were measured. The results showed that there was no significant difference in root length among the three experimental groups (*P* > 0.05, Figure 7A). The seedling height of the Δ*gua*A treatment group did not significantly differ from the other two groups. Still, the seedling height of the group treated with the wild-type strain GXGL-4A t was extremely considerably higher than that of the control group (*P* < 0.01; Figure 7B). There was no significant difference in seedling dry weight between the two NFB treatment groups (*P* > 0.05). Still, both were significantly higher than that of the control group (*P* < 0.05, Figure 7C). There was no significant difference in root dry weight among the experimental groups (*P* > 0.05, Figure 7D).

### 3.8 Changes in the soil enzymatic activity in the cucumber rhizosphere after NFB inoculation

**FIGURE 8:**
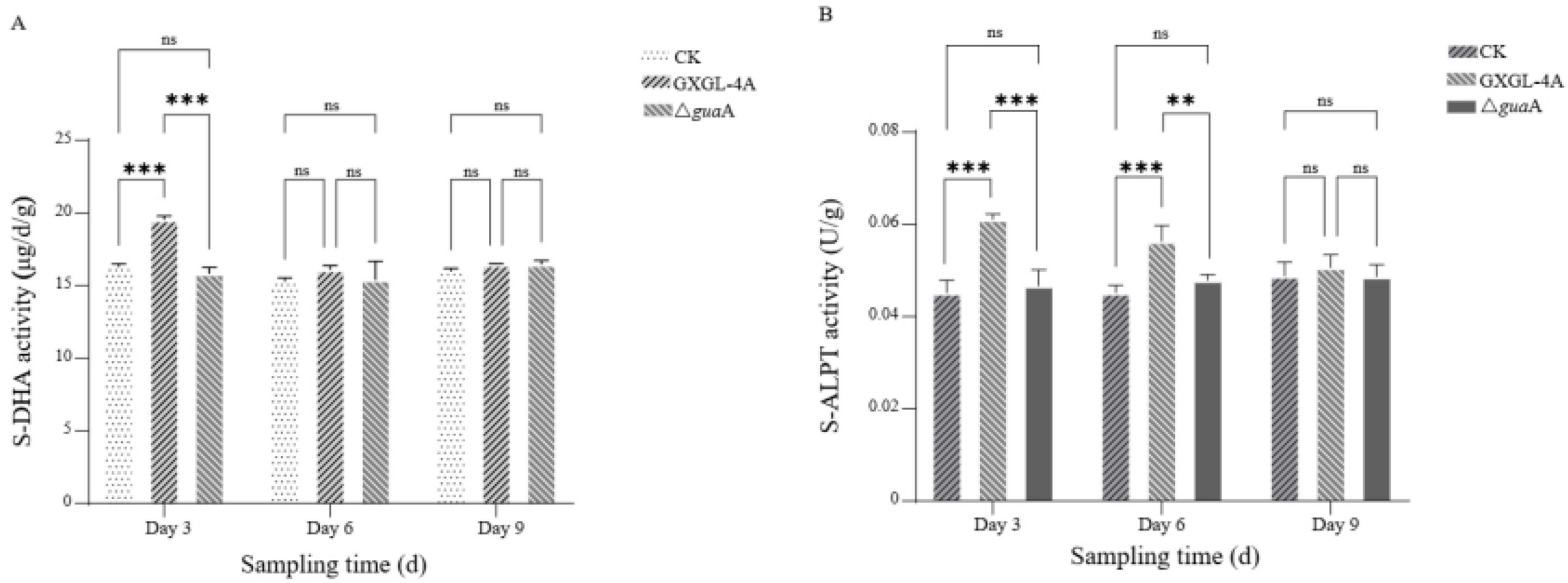
Changes on soil enzyme activity after inoculated with NFB strains. **(A)**: Activities of the S-DHA enzyme on Days 3, 6, and 9 after inoculation with the NFB strains. **(B)**: Activities of the S-ALPT enzyme on Days 3, 6, and 9 after NFB treatment. ns; There is no significant difference in this soil enzyme activity between two groups (*P* > 0.05). **: There is a substainable difference in this soil enzyme activity between two groups at the level of *P* = 0.01. ***: there is a considerable difference in this soil enzyme activity between two groups at the level of *P* = 0.001. CK: The cucumber seedlings without NFB treatment. Δ*gua*A: The cucumber seedlings inoculated with the *gua*A gene deletion strain Δ*gua*A.

The changes in S-DHA and S-ALPT activities in cucumber rhizosphere soil were detected after inoculation with the NFB strains. The results showed that the S-DHA activity of the GXGL-4A treatment group was the highest, and there was an extremely significant difference between the GXGL-4A and the other two experimental groups (CK and Δ*gua*A treatment groups, *P* < 0.01) on Day 3 after NFB inoculation. However, there was no significant difference in S-DHA activity between the CK group and the Δ*gua*A treatment group. On Days 6 and 9 after inoculation with NFB strains, no significant differences in S-DHA enzymatic activity were found between the three experimental groups (*P* > 0.05, Figure 8A). On Days 3 and 6, the S-ALPT enzymatic activities were significantly higher than those in CK and Δ*gua*A groups (*P* < 0.01), but by the 9th day after NFB treatment (Day 9), there was no significant difference in S-ALPT activity between experimental groups (*P* > 0.05). It should be noted that there was no significant difference in S-ALPT activity between the CK and Δ*gua*A groups throughout the experimental period (Figure 8B).

### 3.9 Dynamic change of the cucumber root activity after NFB inoculation

**FIGURE 9:**
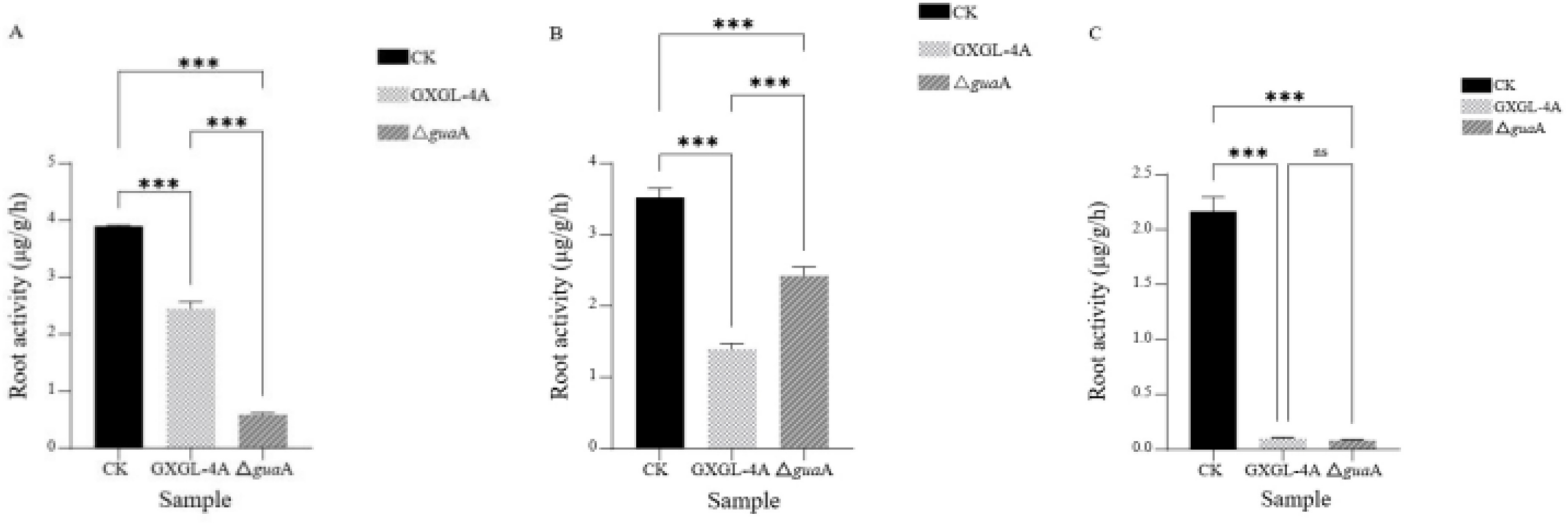
Response of cucumber root activity to inoculated with NFB strains. **(A–C)** Root activity on days 3, 6, and 9 after inoculation. CK: without NFB inoculation; GXGL-4A: inoculated with the wild-type strain GXGL-4A; Δ*gua*A: inoculated with the *gua*A gene deletion strain Δ*gua*A. ***: There is a significant difference in root activity between the two groups at *P* = 0.001. ns: There is no significant difference in root activity between the two groups at the level of *P* = 0.05.

Within 9 days after NFB inoculation, the root activity of the control group (CK group, i.e., non-inoculated with NFB) was significantly higher than that of the NFB treatment groups (*P* < 0.001). Overall, the root activity of both the CK group and the GXGL-4A treatment group gradually decreased. On the 3rd day after inoculation (i.e., Day 3), the root activity of the CK group was the highest, followed by the GXGL-4A treatment group, with the Δ*gua*A treatment group being the lowest. There were highly significant differences among the three experimental groups (*P* < 0.001, Figure 9A). On the 6th day after NFB inoculation (i.e., Day 6), the root activity of the Δ*gua*A treatment group increased significantly compared to the 3rd day. It was extremely substantially higher than that of the GXGL-4A treatment group (*P* < 0.001, Figure 9B). However, by the 9th day after NFB inoculation (i.e., Day 9), the root activity of the Δ*gua*A treatment group was significantly reduced compared to that of the 6th day. Still, there was no significant difference in root activity between the Δ*gua*A treatment group and the GXGL-4A treatment group (*P* > 0.05, Figure 9C). In a word, the root activity fluctuation amplitude of the Δ*gua*A treatment group was significantly greater than that of the other two experimental groups.

## 4 Discussion

The antiSMASH website^1^ allows the rapid genome-wide identification, annotation, and analysis of secondary metabolite biosynthesis gene clusters in bacterial and fungal genomes. It integrates and cross-links with a large number of previously published *in silico* secondary metabolite analysis tools (Kai et al., 2025). According to the online analysis and prediction by antiSMASH (bacterial version 8.0.4), the siderophore biosynthesis gene cluster of the nitrogen-fixing bacterium GXGL-4A is 68,823 nucleotides long (Location in the genome of GXGL-4A: 313,508-382,330) and consists of a total of 56 functional genes (locus tags: A3780_01485 to A3780_01760). The entire gene cluster includes core biosynthetic genes, additional biosynthetic genes, transport-related genes, regulatory genes, and other genes. The antiSMASH prediction results show that *K*. *radicincitans* GXGL-4A can produce *luc*A/*luc*C-like NRPS-independent siderophore (NI-siderophore). The most similar known cluster from MIBiG 4.0 is the xanthoferrin biosynthetic gene cluster from *Xanthomonas oryzae* pv. *oryzae* KACC 10331 (MIBiG accession number: BGC0001408, and location: 1,364,783 –1,397,555 nt) (Pandey and Sonti, 2010). The BLAST alignment results indicated that the siderophore biosynthesis gene cluster of GXGL-4A had an 85% similarity to the gene cluster encoding xanthoferrin, the α-hydroxycarboxylate-type siderophore of *Xanthomonas campestris* pv. *campestris* (Pandey et al., 2017). In this study, the nitrogen-fixing bacterium GXGL-4A has been confirmed to biosynthesize a carboxylate siderophore, and it is unable to synthesize other types of siderophores, as determined by multiple detection methods, including the Shenker test. These results are completely consistent with the bioinformatics prediction results. Furthermore, antiSMASH prediction indicated that the strain GXGL-4A produced siderophore via an NRPS-independent pathway. The absence of the *gua*A gene causes the nitrogen-fixing bacterium GXGL-4A to be unable to produce siderophore. Although the introduction of the *gua*A gene expression vector could promote siderophore synthesis in the Δ*gua*A mutant, the synthesis capacity was not fully recovered. The siderophore content produced in the complemented strain was still significantly lower than that of the wild-type strain GXGL-4A, suggesting that the *gua*A gene is involved in a complex regulatory network controlling siderophore biosynthesis in the bacterium.

For the *gua*A gene deletion strain (Δ*gua*A) that cannot synthesize siderophores, the rational addition of a particular concentration of exogenous guanosine monophosphate can partially restore the strain’s ability to synthesize siderophores. The cyclic dinucleotide c-di-GMP is formed by the condensation of two GTP molecules via diguanylate cyclase (DGC) enzymes. It had been reported that multiple DGCs (DGCs) were involved in the regulation of c-di-GMP on the synthesis of the major iron siderophore pyoverdine in *Pseudomonas aeruginosa* (Chen et al., 2015). Recent research has demonstrated that increasing the concentration of the ubiquitous second messenger c-di-GMP enhances biofilm development, siderophore biosynthesis, and oxidative stress tolerance in *Pseudomonas syringae* (Wang et al., 2023). Intracellular GTP synthase is responsible for synthesizing GTP from GMP and pyrophosphate (PPi), which is the final step in the GTP synthesis pathway. Thus, GTP synthesis is affected by the intracellular concentration of GMP. We deduce that by supplementing with exogenous guanosine monophosphate in culture medium, it can undoubtedly increase the level of intracellular guanosine monophosphate, thereby affecting GTP and subsequently the synthesis of c-di-GMP, ultimately promoting the synthesis of siderophores in the Δ*gua*A strain.

The role of siderophores in the re-greening process has been recently investigated. The siderophore Ferrioxamine E of *Priestia megaterium* ZS-3 could upregulate the expression of chlorophyll genes, thereby increasing photosynthesis and enhancing the transcription of iron and the activity of ferric-chelate reductase in plants. The Arabidopsis thaliana inoculated with the ZS-3 siderophore increased the chlorophyll contents by 3.47-fold (Zhu et al., 2025). In this study, it was shown that cucumber inoculated with siderophore-producing NFB strains could significantly increase leaf chlorophyll content. Once the strain loses the ability to synthesize siderophore, the chlorophyll content in cucumber leaves will dramatically reduce. These results confirm that siderophore treatment can significantly affect the leaf chlorophyll content. As the cucumber plants grew, the chlorophyll content in the leaves gradually decreased. The application of NFB can substantially increase leaf chlorophyll content, especially when inoculated with siderophore-producing NFB strains, resulting in a highly significant increase compared to control plants (*P* < 0.01). The result indicates that the traits of biological nitrogen fixation (BNF) and siderophore production of *K*. *radicincitans* GXGL-4A are beneficial for the enhancement of chlorophyll levels in leaves of cucumber seedlings. There was a report indicating that the inoculation of *Pseudomonas* sp. JIT1 had significantly increased biomass, photosynthesis, and the contents of osmotic substances and chlorophyll in rice seedlings exposed to 2,4,4-trichlorophenol (2,4-DCP) (Wang et al., 2023). Currently, the application of bacterial siderophores primarily focuses on screening plant growth-promoting rhizobacteria and identifying plant growth-promoting and biocontrol traits, including siderophore production and indole-3-acetic acid synthesis (Wu et al., 2025; Nithyapriya et al., 2021). These studies confirm that many rhizosphere-promoting bacteria enhance plant growth, remediate soil pollution by heavy metals, and improve resilience to adverse conditions such as drought and salinity by producing siderophores (Kumar et al., 2021).

The antifungal character of siderophore-producing bacteria has been well studied in recent years. Many bacteria have been shown to exhibit apparent mycelium inhibition against phytopathogens, including *Sclerotium rolfsii*, *Fusarium oxysporum*, *Botrytis cinerea*, *Colletotrichum gloeosporioides*, and *Rhizoctonia solani*. The bacterial siderophore has been evaluated as a possible biocontrol mechanism (Mahebub et al., 2025; Lakshmikanthan et al., 2025). *B*. *amyloliquefaciens* SS7 exhibited the highest siderophore production and vigorous antifungal activity. Research has shown that bacterial isolates with high siderophore production capabilities exhibit potent antifungal activity (Kumar et al., 2025). *K*. *radicincitans* GXGL-4A exhibited specific inhibitory effects on the growth of hyphae of several phytopathogenic fungi, especially showing good antagonistic activity against the fungi *R*. *solani* and *B*. *cinerea*. Compared to the wild-type strain GXGL-4A, the antibacterial activity of the deletion strain Δ*gua*A was significantly reduced, which may be due to the loss of the ability to synthesize siderophore.

Soil electrical conductivity (EC) is an indicator of soil-soluble salts and a factor in determining whether soil salt levels limit crop growth. EC value of the soil extract can reflect the content of soluble salts in the soil, thereby evaluating the degree of soil salinization. Generally, the higher the EC value, the higher the soil salt concentration. Soils from salt-affected regions were found to have high electrical conductivity (EC) and pH levels, along with low nutrient availability (Tiwari et al., 2024). In this study, the EC values of vermiculite leachate in the rhizosphere of cucumber in each experimental group showed a clear and continuous downward trend. This may be because plants absorb nutrients and water from the substrate during growth, thereby reducing soil salt concentration and, consequently, decreasing the EC value. The rhizosphere of cucumber seedlings inoculated with the ΔguaA strain showed the lowest EC, suggesting its promising potential to alleviate salt stress in cucumber under a nitrogen-free vermiculite cultivation medium. The pH of the rhizosphere environment in the two NFB treatment groups remained stable at weakly alkaline levels (7.35–7.71). In contrast, the control group’s pH was weakly alkaline during the early and mid-experiment stages (Days 3 and 6), but became weakly acidic (pH 6.45) by the end of the experiment (Day 9). We speculate that NFB strains convert atmospheric nitrogen into ammonia via biological nitrogen fixation (BNF), thereby maintaining a weakly alkaline rhizosphere in the NFB treatment groups. In contrast, the control group, which was not inoculated with exogenous strains, showed changes in pH due to the continuous accumulation of acidic components, such as small organic acids, in root exudates, ultimately resulting in a weakly acidic rhizosphere environment. In a word, the NFB strain GXGL-4A showed potentially attractive attributes like siderophore production, antifungal activity, and plant growth-promoting traits. Further comprehensive studies should be conducted to explore this bacterium under field conditions.

## 5 Conclusion

This study analyzed the siderophore type synthesized by *K*. *radicincitans* GXGL-4A and determined the effects of NFB siderophore-producing capability on cucumber plant growth and its antiphytopathogenic fungal activity. The results showed that the strain GXGL-4A could produce a hydroxycarboxylate-type of siderophore. The ability of the *gua*A gene knockout strain (Δ*gua*A) to synthesize siderophores can be partially restored by supplementing 2% (W/V) guanosine monophosphate in LB medium. If the nitrogen-fixing bacterium GXGL-4A loses its ability to produce siderophores, it will significantly reduce its own antifungal capacity and the growth-promoting effect on cucumber plants. The dry weight of cucumber seedlings, root dry weight, and leaf chlorophyll content after inoculation with the strain Δ*gua*A were significantly lower than those in the wild-type strain GXGL-4A treatment group. However, there was no significant effect on root activity, soil dehydrogenase, and alkaline protease activity between the GXGL-4A and the Δ*gua*A treatment groups. Inoculation with nitrogen-fixing bacteria helps maintain relatively stable electrical conductivity, pH, and root activity in the cucumber rhizosphere.

## Data Availability

The datasets presented in this study can be found in online repositories. The names of the repository/repositories and accession number(s) can be found below: https://www.ncbi.nlm.nih.gov/genbank/, CP015113.1.

## Footnotes

1
https://antismash.secondarymetabolites.org/
